# OR6-005 – Cystine crystals activate inflammasomes

**DOI:** 10.1186/1546-0096-11-S1-A100

**Published:** 2013-11-08

**Authors:** G Prencipe, I Caiello, S Petrini, L Bracci Laudiero, F Emma, F De Benedetti

**Affiliations:** 1 Laboratory of Rheumatology, Bambino Gesù Children's Hospital, Roma, Italy; 2 Department of Nephrology and Urology, Bambino Gesù Children's Hospital, Roma, Italy; 3 Operative Unit of Rheumatology, Bambino Gesù Children's Hospital, Roma, Italy

## Introduction

Nephropathic cystinosis is a rare autosomal recessive disorder caused by a mutation in the CTNS gene, which encodes for cystinosin. It is characterized by the lysosomal accumulation of cystine, which leads to the formation of cystine crystals within various organs, including kidneys, brain, cornea, intestine and bone marrow. The exact role of intralysosomal cystine crystals accumulation in the pathogenesis of clinical features of cystinosis is still unclear, although it is well known that cystine levels are directly proportional to disease severity.

## Objectives

In this study, we investigate whether cystine crystals are able to elicit inflammasome activation.

## Methods

Primary human peripheral blood mononuclear cells (PBMCs) were cultured in vitro, pre-incubated with LPS, stimulated with L-cystine crystals in presence or absence of different inhibitors and the IL-1beta (IL-1b) released in the medium was measured by ELISA.

## Results

LPS-primed PBMCs stimulated with L-cystine crystals secreted IL-1b in a dose-dependent manner. Similarly to other NLRP3-activating particles, cystine crystal-induced IL-1b secretion was caspase-1-dependent. Indeed, when PBMCs were pre-icubated with the specific CASP-1 inhibitor (Z-YVAD-fmk), a dramatic decrease in IL-1β production was observed, suggesting the involvement of an inflammasome-mediated pathway. By confocal microscopy, we observed that exogenous L-cystine crystals were internalized by monocytic/macrophagic adherent cells. Inhibition of actin polymerization with cytochalasin D effectively blocked cystine crystal-induced IL-1β secretion, showing that phagocytosis is necessary for this effect.

## Conclusion

Taken together, these data demonstrate that cystine crystals represent a new endogenous inflammasome activating danger signal, suggesting a new role for cystine crystals in the pathogenesis of nephropathic cystinosis.

## Disclosure of interest

None declared

